# Comparison of informational vs. energetic masking effects on speechreading performance

**DOI:** 10.3389/fpsyg.2014.00639

**Published:** 2014-06-24

**Authors:** Björn Lidestam, Johan Holgersson, Shahram Moradi

**Affiliations:** ^1^Department of Behavioral Sciences and Learning, Linköping UniversityLinköping, Sweden; ^2^Linnaeus Centre HEAD, Swedish Institute for Disability Research, Department of Behavioral Sciences and Learning, Linköping UniversityLinköping, Sweden

**Keywords:** speech perception, cognition, speechreading, informational masking, energetic masking

## Abstract

The effects of two types of auditory distracters (steady-state noise vs. four-talker babble) on visual-only speechreading accuracy were tested against a baseline (silence) in 23 participants with above-average speechreading ability. Their task was to speechread high frequency Swedish words. They were asked to rate their own performance and effort, and report how distracting each type of auditory distracter was. Only four-talker babble impeded speechreading accuracy. This suggests competition for phonological processing, since the four-talker babble demands phonological processing, which is also required for the speechreading task. Better accuracy was associated with lower self-rated effort in silence; no other correlations were found.

## Introduction

In everyday speech perception, we hear speech clearly and without effort. Speech perception is usually pre-dominantly auditory. We might see the person talking, and this may help us perceive the speech more distinctly, especially if the speech signal is degraded or masked by noise (e.g., Hygge et al., 1992; Calvert et al., 1997; Moradi et al., 2013), but often the acoustic speech signal is enough for us to hear what is spoken. For most people, hearing speech is usually effortless and efficient. We are able to perceive a sufficient proportion of the speech sounds for comprehension of the speech signal.

Occasionally a speech signal will be masked by noise (i.e., sounds other than the voice of the person we are trying to hear). There are two main ways that noise can interfere with the speech signal. First, the noise can physically interfere with the speech signal (i.e., outside of the perceiver, in the acoustic environment). This is often referred to as *energetic masking* (Pollack, 1975). Second, the noise can perceptually interfere with the speech signal (i.e., inside the perceiver, in the perceptual process). This is often referred to as *informational masking* (Pollack, 1975; Watson et al., 1976).

Disentangling informational masking from energetic masking in auditory perception is difficult, as can be seen in the literature (e.g., Watson, 2005; Yost, 2006; Kidd et al., 2007). How can the detrimental effect of noise on speech perception be attributed to either informational masking, or to energetic masking (or to attentional allocation as a result of stimulus degradation)? Obviously, if an acoustic speech signal is presented together with an acoustic noise signal, there will necessarily be some degree of energetic masking. Elaborate study designs (such as that in Mattys et al., 2009) are required to dissociate the two types of masking.

The present study solved this problem by *not* presenting an acoustic speech signal, and by instead testing the effect of two closely matched types of noise (henceforth referred to as *auditory distracters*) on visual-only speechreading. That is, there was no possibility of energetic masking, as there was no acoustic signal to mask, interfere with, or compete with. Any effects of the auditory distracter could therefore be attributed to either attentional or phonological processing, or to a combination of both.

In order to test whether there was a general effect on attention, a broadband *steady-state noise* (SSN) was used. As the SSN does not contain phonological information, its potential effect on speechreading performance (i.e., linguistic processing) is likely to be indirect. Specifically, more attentional resources might be needed for stream segregation, leaving less for the search of critical visual speech features when trying to make a lexical match, thereby lowering speechreading performance. Alternatively, SSN could improve speechreading performance via stochastic resonance, whereby a signal (such as auditory noise) in one modality can facilitate perception in another modality (see e.g., Harper, 1979; Manjarrez et al., 2007; Söderlund et al., 2007; Lugo et al., 2008; Ward et al., 2010; Tjan et al., 2013; Gleiss and Kayser, 2014).

In order to test whether there was an effect on phonological processing, segmented *four-talker babble* (4TB) was used. The 4TB was matched to the broadband SSN in terms of average sound intensity and frequency. The 4TB is speech, and so contains phonological information. Any effect of the 4TB on speechreading performance will therefore be more complex. There could be a general effect on attention and stream segregation, or facilitation from stochastic resonance, similar to effects of the SSN. There could also be an effect of competition for phonological processing capacity (i.e., identifying the babble sounds as speech and making lexical matches), while simultaneously having to decode the visual speech movements as phonemes in order to make a lexical match.

Lower speechreading performance from the 4TB condition compared to the SSN condition would indicate an effect on phonological processing. Both auditory distracter types contained equivalent levels of acoustic energy across the frequency spectrum and should therefore affect general attention or induce stochastic resonance to a similar extent. However, the 4TB also requires phonological processing, whereas the SSN does not. Brungart and Simpson (2005) found that visual-only identification of a word was impeded only by simultaneous auditory presentation of another word (spoken by one talker). They therefore suggested that only *speech* as auditory distracters can impair visual-only speechreading (since other distracters yielded no effects). Furthermore, auditory distracters must according to Brungart and Simpson (2005) be presented simultaneously with the visual speech (as synchronicity reduced the impairment).

In the present study, we wanted our visual speech identification task to be as free from contextual cues as possible, as has been the case in most studies on auditory speech perception in noise. In studies on auditory speech perception, the standard case is to allow the participants to perceive relatively clearly what is being said; the acoustical speech signal is rich in information and can be effortlessly identified without contextual cues. This is usually *not* the case for visual-only speechreading, since the optical speech signal is poorly defined (as compared to standard acoustical speech signals). We wanted our speechreading task to be primarily processed bottom up. That is, we wanted it to be a context-free (or non-primed) visual speech identification task, and for two reasons. First, we wanted to have high external and ecological validity, that is, to make the speechreading task as similar to everyday speech perception as possible (e.g., like watching someone talk behind a window pane or seeing someone talk on TV with the sound turned off; in real life we usually do not get closed sets of response alternatives). Second, we wanted the speechreading task to demand high sensitivity to phonological features to allow phonemic–lexical matches, with little influence from top-down support, in order to maximize chances for the auditory distracters to disturb speech identification.

However, it is not possible to use such a bottom-up task with a normal population without obtaining floor effects, since optical speech signals are poorly defined. Most individuals do not perform above chance levels on visual speech decoding tasks unless there is strong contextual support for top-down inferences, such as from script (e.g., Samuelsson and Rönnberg, 1993), topic (e.g., Hanin, 1988), emotional cues (e.g., Lidestam et al., 1999), or a closed set of response alternatives (e.g., coordinate response measure, Brungart and Simpson, 2005). Including strong contextual cues or having a closed set of response alternatives can improve speechreading performance to relatively high levels for a cross-section of normal-hearing participants. However, such improved accuracy is not necessarily the result of more efficient lexical processing. If sufficient contextual cues are available, it is possible that responses are based on post-lexical inferences rather than on actual lexical matches. Hence, a substantial proportion of the responses (made following the presentation of strong contextual cues) may reflect educated guesses (“off-line” responses) rather than improved perceptual accuracy (“on-line” responses). In order to maximize the chances for linguistic (phonemic–lexical matching) processing in visual speechreading, we screened a relatively large number of individuals, and used only the best-performing speechreaders in the actual experiment, asking them to speechread everyday words without contextual cues.

This study aimed to shed more light on informational masking (i.e., disturbed speech perception) by contrasting two different auditory distracters: speech (i.e., the 4TB) compared to SSN. The 4TB was a continuous stream of speech, and was therefore not presented synchronously with the target words, as was the case in Brungart and Simpson (2005). As a baseline condition, speechreading in silence (i.e., without auditory distracter) was used. Effects of SSN could only be attributed to general attentional processes, as SSN does not contain phonological information. On the other hand, 4TB, with speech as an auditory distracter, contains phonological information. Any difference between SSN and 4TB can therefore be attributed to impeded visual phonemic–lexical matching elicited by the 4TB distracter signal. A negative effect of either type of auditory distracter would suggest that synchronicity is not required to impair speechreading accuracy. A positive effect on speechreading performance would suggest facilitation from stochastic resonance.

A secondary purpose of the study was to examine how the auditory distracter conditions were subjectively experienced in terms of level of distraction, effect on performance, and effort, to validate the effects on speechreading accuracy.

Finally, this study aimed to test whether there were correlations between self-rated variables and speechreading performance, in order to aid interpretations of how attention and phonological processing were affected by the auditory distracter conditions.

## Screening test

### Methods

#### Participants

A total of 147 students at Linköping University (90 women, 53 men, and 4 who did not divulge sex and age), aged 18–37 years (*M* = 21.6 years, *SD* = 2.8 years), volunteered to take part in the study.

#### Materials

The stimulus materials were video recordings of the best identified 30 words as used in the study by Lidestam and Beskow (2006). Half of the words were from a “visit to a doctor” script, and half were from a “visit to a restaurant” script. The recordings showed a man speaking one word at a time, with a neutral facial expression. The words consisted of three to seven letters (and phonemes), with one or two syllables. All words were rated as highly typical for their respective script. The presentation showed the talker's face and shoulders, and no shadows obscured the mouth or speech movements. For a detailed description, see Lidestam and Beskow (2006).

#### Procedure

The screening test was conducted in lecture halls. The stimuli were presented with video projectors onto either one or two (if available) large screens. After written informed consent was obtained, the participants positioned themselves within the lecture hall in such a manner that the screen was easily visible. They were encouraged to sit so they would not be able to see other participants' response sheets. After seating, they were provided with response sheets and pencils, and informed about the general purpose of the study. Specifically, the participants were informed that the study was about speechreading and that this first part was a screening test for an experiment that would be more exhaustive and rewarded with a cinema ticket. It was made known that only the best speechreaders would be invited to take part in the main study, if they agreed to do so (participants indicated their willingness by checking a box on the response sheet).

The participants were instructed that their task was to speechread (without sound) the words spoken in two scripts: “a visit to a doctor” and “a visit to a restaurant.” It was stated clearly that there was no hidden agenda, and that it was important to try their best to guess and to respond to all stimuli. They were also informed that the responses did not need to be whole words, and that parts of words were preferred as responses over no response at all, but that if only a part of a word was rendered (e.g., a consonant), its position in the word should be indicated.

Stimuli were presented in two script blocks. Before presentation of each block, the respective scenario was presented with text on the screen. The words were then presented at a reasonable pace that allowed all participants to respond without undue stress. The screen was black in between presentation of the words. At the end of the screening sessions, the participants indicated whether they could be contacted for the experiment that would follow. In total, the screening session took 20 min. After the session, the participants were given the opportunity to ask questions and were offered refreshments.

The responses for phonetic correctness were scored on a whole word basis; that is, each word was scored dichotomously as either correct or incorrect. Omissions or inclusions of word endings with /t/ were disregarded (e.g., “normal” vs. “normalt” [normal vs. normally]; “dåligt” vs. “dålig” [bad vs. badly]).

### Results and discussion

Mean speechreading performance in the screening test was *M* = 2.2 words (*SD* = 2.55 words, range 0–12 words). Fifty-two percent of participants responded with zero or only one word correct. Out of the 147 participants in the screening test, 130 agreed to be contacted for the experiment. Their mean score was *M* = 2.2 words (*SD* = 2.48 words, range 0–12 words).

These results show that visual-only speechreading is a difficult task for most individuals. Just over half of the participants correctly identified 0 or 1 word out of 30. However, the top performers (the best 5%) could identify as many as one-third of the words (but this came as a surprise to them when told about their results). This shows that there is considerable variability in the population of normal-hearing young students with regard to speechreading ability.

## Main experiment

### Methods

#### Participants

All participants who achieved a total score of three or more on the screening test and who had indicated on the scoring sheets that they could be contacted for participation in the main experiment (*n* = 43) were asked to participate. Potential participants were informed that normal hearing was a requirement, and that their participation would be rewarded with a cinema ticket. A total of 23 students (21 women and 2 men), with a mean age of 21.9 years (*SD* = 2.7 years, range 19–31 years), participated in the experiment.

#### Materials

The stimuli were video recordings of a woman speaking a selection of the 5000 most common Swedish words in everyday use (according to the Swedish Parole corpus; Språkbanken, n.d.). The talker's face and shoulders were shown, and indirect lighting was used so that no shadows obscured the speech movements.

We wanted to use common, everyday Swedish words that were relatively easy to speechread, even without contextual cues. The words were therefore chosen according to the following criteria. First, each word had to be ranked among the 5000 highest frequency Swedish words according to the Parole corpus. Second, variation with regard to the number of syllables was considered; hence, words with one to five syllables were used. Third, the majority of the stimulus words contained consonants that are relatively easy to identify visually, and preferably in initial position. Before deciding which words to use, all candidate words were scored for visual distinctiveness according to whether any of the visually distinct consonants /f v b m p/ were part of the word, and a bonus score was given if the visually distinct consonant was in initial position (i.e., the first or second phoneme). The score was then normalized by dividing the sum of the scores for visually distinct consonants and bonus scores for initial position by the total number of phonemes in the word. A total of 180 words were chosen using this procedure. The words were divided into three different lists with 60 words in each. The lists were balanced in terms of: visual distinctiveness, word frequency (according to the Parole corpus), initial phoneme, and number of phonemes per word (Supplementary Material).

A Sony DCR-TRV950 video camera was used to record the stimuli to mini-DV tape in PAL standard at 25 frames per second. Each stimulus word was recorded twice and the best recording of each word was chosen. The recording was edited into separate QuickTime files, one per stimulus word, in H.264 video format at 640 × 480 pixels. Only the video track was exported, in order to eliminate the risk of speech sound being presented. Each video file was edited so that the first frame was repeated for 25 frames (i.e., 1 s) before the actual playback of the video. (This was done in order to cue the participant to the presentation, and to minimize the risk of failure to play back at the correct frame rate due to processing demands, as video playback tends to lag within the first second when using standard software such as QuickTime for playback).

Each stimulus file was then edited into one new file per condition. The files for the baseline condition in silence were kept without sound, whereas each file for presentation in the SSN condition included a unique part of the SSN, and each file for the 4TB condition included a unique part of the 4TB.

**Figure 1:**
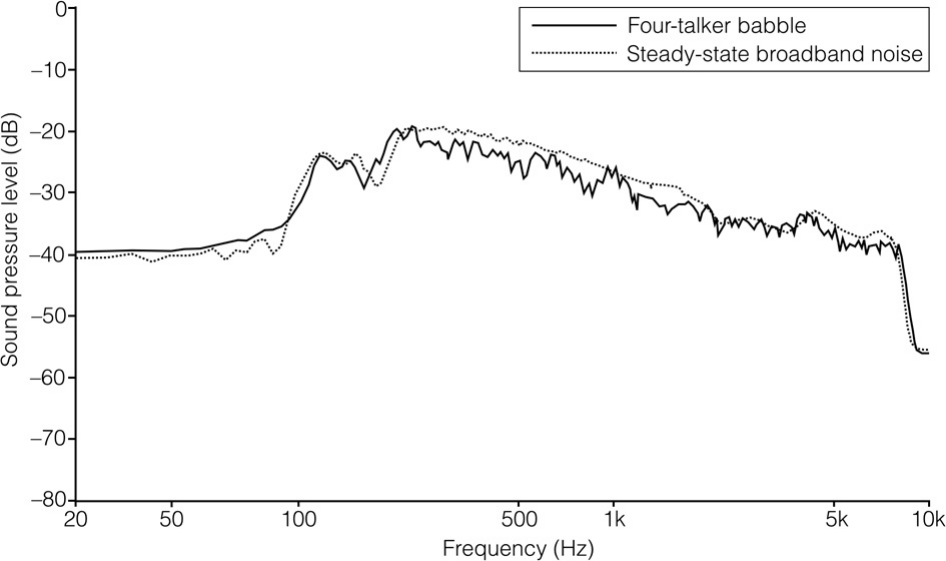
**The long-term average spectrum for the two auditory distracters**.

**Figure 2:**
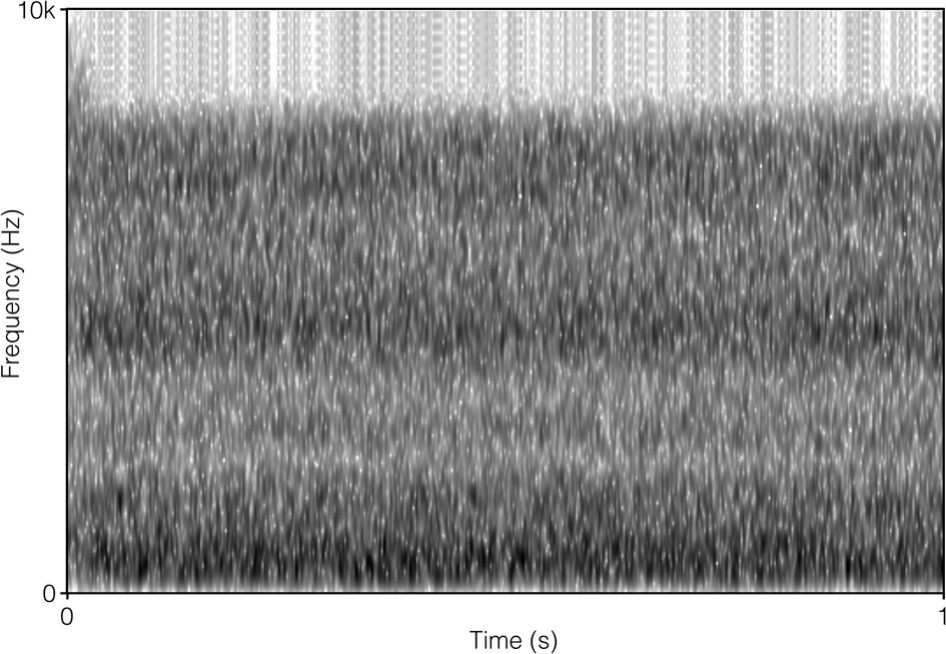
**Spectrogram of 1 s of steady-state broadband noise**.

**Figure 3:**
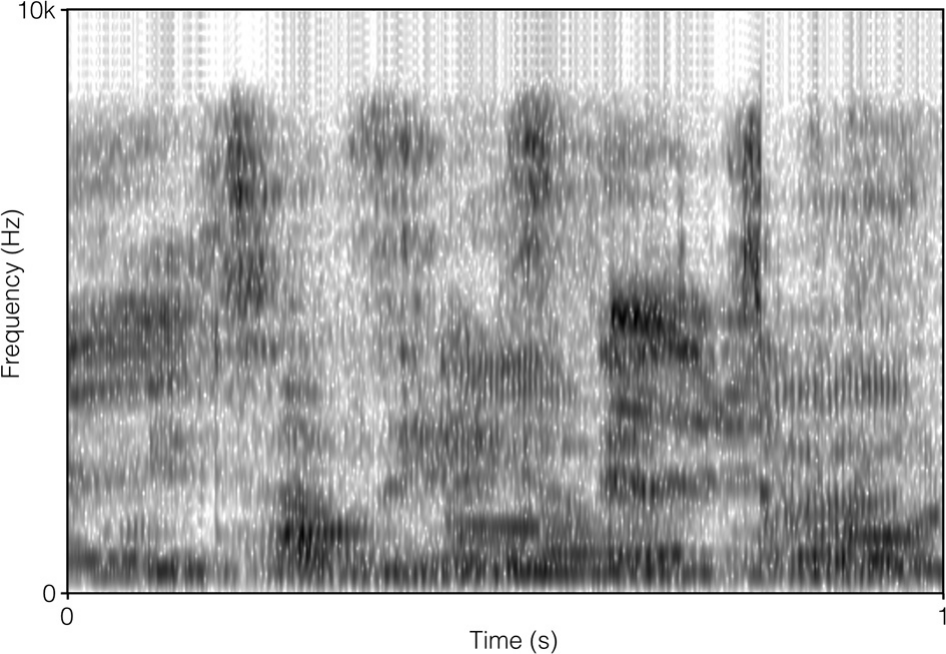
**Spectrogram of 1 s of four-talker babble**.

The SSN was the stationary, speech-shaped broadband noise used in the Swedish Hearing in Noise Test (HINT; Hällgren et al., 2006), and has the same long-term average spectrum as the HINT sentences. The original file with the 4TB was 2 min in duration, and comprised recordings of two male and two female native Swedish talkers reading different paragraphs of a newspaper text. It was post-filtered to resemble the long-term average spectrum of the HINT sentences (Ng et al., 2013). In order to prevent participants from directing their attention to the content of the 4TB sentence (which was a finding suggested by the pilot study), the file was cut up into approximately 0.5 s sections, and scrambled so that the order of sections 1, 2, 3, 4, 5, 6 became 1, 3, 2, 4, 6, 5, and so on. Pilot testing verified that this was well tolerated by participants. It also indicated that the stimuli no longer roused attention regarding content. There were no apparent clicks resulting from the editing. For a comparison of the long-term average spectrum of the two auditory distracter types, see Figure 1. For a comparison of the spectral-temporal contents of the two auditory distracter types over a segment of 1 s, see Figure 2 (SSN) and Figure 3 (4TB).

The apparatus for presentation included an Apple iMac 8.1 computer with a 2.4 GHz Intel Core Duo processor, 2 GB RAM, and an ATI Radeon HD 2400 XT with 128 MB VRAM. A 20-inch monitor (set at 800 × 600 pixels), Primax Soundstorm 57450 loudspeakers (capable of 80–18,000 Hz), and Tcl/Tk and QuickTimeTcl software were used to present the stimuli.

A Brüel and Kjær sound level meter type 2205 with a Brüel and Kjær 1 inch free-field microphone type 4117 were used to monitor sound pressure levels of the auditory distracters. These were placed at the approximate position of the participants' ears. Both auditory distracter types had equivalent continuous A-weighted sound pressure levels (LAeq) of 61 dB (SSN range = 59.7–62 dB; 4TB range = 52.4–70 dB) for the 2 min measurement during which the entire auditory distracter files were presented.

In order to examine how the auditory distracter conditions were subjectively experienced in terms of level of distraction, effect on performance, and effort, two questionnaires with 100 mm visual analogue scales were used. Scoring was calculated according to how many millimeters from the minimum (0 mm) the scale was ticked by the participants; hence maximum score was 100 mm.

#### Procedure

Each participant was seated in front of the monitor at a distance of approximately 60 cm. They were briefed about the general purpose of the study (i.e., they were informed that their task involved speechreading under three different sound conditions), and written informed consent was obtained. A response sheet with numbered lines for each presented stimulus was introduced, and the participant was instructed to respond to all presented words and encouraged to guess. Then a recording of a word that was not included in the actual experiment was presented, with the same auditory distracter condition as the participant started the experiment with, to familiarize the participant with the procedure.

The stimuli were presented one at a time; the speed of presentation was dictated by the pace of participant responding, but there was a maximum limit of 1 min (which never needed to be used). The screen turned white in the pause between stimuli. For all three conditions (i.e., silence, SSN, and 4TB), the sound continued during the pause (i.e., in the silent condition, the pause was silent too; in the SSN condition, the SSN continued during the pause; and in the 4TB condition, the 4TB continued during the pause).

Scoring followed the procedure used in the screening test, such that the responses were dichotomously scored for phonetic correctness on a whole word basis.

After performing in each auditory distracter condition, the participant filled out the subjective experience questionnaire (which concerned experiences of each sound condition, self-ratings of performance, and which included some open-ended questions; see Supplementary Material). At the end of the experiment, the participants were awarded with a cinema ticket as reward for participation, and were given the opportunity find out more about the experiment. The experimental session took approximately 50 min to complete.

#### Design

This study employed a within-groups design, with auditory distracter as the independent variable (three levels: silent, SSN, and 4TB), and speechreading accuracy as the dependent variable. A Latin-square design was used to determine the presentation orders of conditions (silence, SSN, and 4TB) and lists (1–3), so all experimental conditions and lists were combined and presented in all serial positions. Participants were randomized to presentation orders.

### Results

#### Effect of auditory distracter on speechreading accuracy

Auditory distracter significantly affected speechreading accuracy, *F*_(2, 44)_ = 11.19, *MSE* = 6.21, *p* < 0.001, partial η^2^ = 0.34. Three *post-hoc t*-tests with Bonferroni corrected alpha (*p* < 0.17) showed a significant difference between the 4TB and silence conditions, *t*_(22)_ = 2.98, *p* = 0.007, *d* = 0.62, and between the 4TB and SSN conditions, *t*_(22)_ = 4.65, *p* < 0.001, *d* = 0.97. There was no significant difference between the SSN and silence conditions, *t*_(22)_ = 1.71, *p* = 0.101. In sum, only 4TB had an effect on speechreading accuracy, and this effect was negative (see Table 1).

**Table 1:** **Descriptive statistics (means and standard deviations) for accuracy, and self-ratings of effort, performance, distraction, and effect of auditory distracter on performance**.

	**Auditory distracter condition**					
	**Silence**		**SSN**		**4TB**	
	**M**	***SD***	**M**	***SD***	**M**	***SD***
Accuracy (% correct)	14.8	6.6	16.9	6.2	11.2	5.9
Self-rated effort (0–100)	46.6	24.3	38.0	19.7	32.8	17.8
Self-rated performance (0–100)	42.5	15.1	44.1	18.3	37.9	16.1
Rated distraction of auditory distracter (0–100)	n.a.	n.a.	40.7	23.0	45.3	22.6
Rated effect of auditory distracter on performance (0–100)	n.a.	n.a.	40.2	16.7	32.7	18.0

Most error responses were words that included one or several correct phonemes, and one or several incorrect phonemes (about 75% of all responses belonged to this category). The second most common errors were words without any correct phoneme (and the majority of these errors were words with one or more phonemes which were easily visually confused with phonemes in the target word, such as /f/ instead of /v/ or /b/ instead of /p/). The third most common error was failure to respond with a proper word, such as only responding with a few letters as a part of a word. Omissions (i.e., no response at all to a target word) constituted the least common cause of errors, with 8% of the total number of responses.

#### Effects of auditory distracter on ratings of effort, distraction, and performance

Auditory distracter had a significant effect on participants' self-ratings of effort, *F*_(2, 44)_ = 3.40, *MSE* = 3.30, *p* = 0.042, partial η ^2^ = 0.13. Three *post-hoc t*-tests with Bonferroni correction (*p* < 0.17) revealed only a tendency toward a significant difference between the silence and 4TB conditions, *t*_(22)_ = 2.32, *p* = 0.03. The means (see Table 1) indicate that the speechreading task was generally perceived as effortful, and that speechreading in the 4TB condition was considered to be very effortful. Comparisons between the two auditory distracter conditions indicated no effect of auditory distracter type on rated distraction or self-rated performance. The mean ratings suggested that participants considered both types of auditory distracter to have impeded their performance to a considerable extent; both types of auditory distracter were rated as more toward “almost unbearable” than “not distracting at all.”

#### Correlations between speechreading accuracy, and ratings of effort, distraction, and performance

Table 2 presents the correlation results. The only significant correlation was between accuracy and self-rated effort in the silence condition, *r*_(23)_ = 0.44, *p* < 0.05. Specifically, better performance was associated with lower effort ratings in the silent condition (high scores on the self-rating indicated low effort). However, there was no difference between the correlation coefficients for silence vs. 4TB.

**Table 2:** **Pearson correlations between accuracy and ratings**.

	**Auditory distracter condition**		
	**Silence**	**SSN**	**4TB**
Self-rated effort	0.44^*^	0.00	0.17
Self-rated performance	0.11	0.22	−0.04
Rated distraction of auditory distracter	n.a.	−0.01	−0.15
Rated effect of auditory distracter on performance	n.a.	−0.01	0.09

* p < 0.05.

## Discussion

The present study showed that visual-only speechreading was only impeded by an auditory speech-based distracter, but not by noise itself. This implies that in order for the distracter to have an impact, it has to compete for phonological processing, which is required for identification of the visual speech signal. More “general” auditory distraction, such as the SSN stimuli used in this study, did not impede speechreading accuracy, in spite of that it was rated as very distracting by the participants. Competition for phonological processing (and following semantic processing) demands processing related to working memory, such that individuals with superior working-memory related capacities are less impeded by speech and speech-like distracters (e.g., Rönnberg et al., 2010; Zekveld et al., 2013). Markides (1989) showed an effect of classroom noise (including some speech sounds) on visual-only speechreading performance, but it is likely that the frequent and intermittent peaks of the noise (up to 97.5 dBA) interfered with attention as a result of their unpredictability and sheer sound pressure level—it is difficult not to be distracted by such loud sounds.

The participants in the present study were above-average speechreaders recruited among normally hearing students. Speechreading performance is positively correlated with aspects of working memory in this population (Lidestam et al., 1999). Therefore, the impediment effect on speechreading by a speech-based auditive distracter should be potentially even stronger on the majority of normally hearing individuals, since they generally have lower working-memory related capacities (Lidestam et al., 1999) and are more impeded by speech and speech-like distracters (e.g., Rönnberg et al., 2010; Zekveld et al., 2013). Individuals who are less proficient speechreaders also perceive the visual speech as very indistinct, making them even more disadvantaged (i.e., the weaker the percept, the easier to disrupt it).

The present study also showed that the auditory distracter signal does not need to be simultaneous in terms of onset relative to the visual speech signal, as suggested by Brungart and Simpson (2005). The auditory speech signal in the present study was four-speaker babble and was therefore more or less continuous.

Energetic masking can be ruled out as an explanation of impeded speech identification in this study, as there was no acoustic speech signal and hence no sound energy for the distracter signal to interfere with. Thus, the effect of the distracters on speechreading accuracy appears to have been purely “informational.”

No facilitation from either auditory distracter was found, but this should be further investigated in studies with more statistical power and higher sound pressure levels for SSN (in order for facilitation from stochastic resonance 70–80 dB is recommended; see e.g., Harper, 1979; Usher and Feingold, 2000; Manjarrez et al., 2007; Söderlund et al., 2007). The results from the present study suggest strongly that auditory speech distracters, such as 4TB, cannot facilitate speechreading, and it is unlikely that facilitation would occur under any sound pressure level. Many studies on auditory speech perception have found that speech and speech-like distracters, such as speech-shaped modulated noise, impede identification of speech targets (e.g., Festen and Plomp, 1990; Hygge et al., 1992; Hagerman, 2002; George et al., 2006; Zekveld et al., 2013).

As visual-only speech signals are generally poorly defined, almost any auditory distraction could potentially have a negative effect on the detection and identification of the subtle features of the speech movements involved. However, some previous studies failed to find effects, even of speech as distracter, on visual-only speechreading performance, except when the distracter signal was similar to the targets and presented synchronously (Brungart and Simpson, 2005; see also Lyxell and Rönnberg, 1993). In the Brungart and Simpson (2005) study, a coordinate response measure task was used in the condition where an effect of auditory distracters was found; this task has limited response alternatives. Further, the distracter signal was a simultaneous auditory presentation of one talker speaking one of the few response alternatives to the visual target. Therefore, the task in that study can be assumed to have been more demanding in terms of attentional allocation and stream segregation (as there was only one talker, and the onset of the auditory distracter word was synchronized to the onset of the speech movements). For that reason, Brungart and Simpsons' effect of phonological interference is more difficult to interpret than the findings of the present study. The generalizability to everyday speech perception of the results from the present study can also be claimed to be higher compared to the results in Brungart and Simpsons' study, since everyday communication does not often provide such closed sets of response alternatives or situations resembling coordinate response measure tasks.

The hypothesis that average and below-average speechreaders should be more disturbed by auditory speech distracters, compared to above-average speechreaders, would require a highly structured task, such as a coordinate response measure task or stimuli that are extremely visually well defined, however. Floor effects would be difficult to avoid otherwise: if performance is at the floor at baseline it cannot decrease.

The only significant correlation found was between speechreading accuracy and self-rated effort in the silent condition (i.e., without auditory distracter). This finding may indicate that segregating the speech (the speech movements, the phonological information that the speech movements elicit, or both) from the distracter signal (i.e., the SSN or 4TB) increased the cognitive load, which made the ratings less accurate. That is, it is possible that there was not enough cognitive spare capacity to accurately rate own effort after segregating speech from noise, which would mean that the task was more cognitively demanding than realized by the participants. This explanation is in line with the conclusions from studies suggesting that segregating input from different signal sources requires cognitive effort (e.g., Mishra et al., 2013; Zekveld et al., 2013).

### Conflict of interest statement

The authors declare that the research was conducted in the absence of any commercial or financial relationships that could be construed as a potential conflict of interest.
