# Supplementary material for: Comparison of informational vs. energetic masking effects on speechreading performance
Source: Front Psychol. 2014 Jun 24;5:639. doi: 10.3389/fpsyg.2014.00639 (PMC4068195; doi:10.3389/fpsyg.2014.00639)
Supplement: Supplementary file 1 [file Presentation1.PDF]

Appendix A: Words used in the main experiment

Presentation orders

List 1	List 2	List 3
prov	varm	vit
affär	timme	professor
försvar	viktigaste	finna
båt	fisk	begreppet
befolkningen	nivå	tempo
efterfrågan	spela	medvetet
morfar	försvunnit	stämma
tuffa	afrika	medarbetare
smula	förhandlingarna	eftermiddagen
värderingar	ifall	förvandlas
musikaliska	häromdagen	riva
iväg	bevis	mil
berg	mormor	plan
sommar	uppgiften	författare
omfattar	klubb	färg
ekonomiska	lämna	massa
överallt	proffs	visserligen
uppmärksammade	förhoppningar	summa
demokrati	män	förlust
kombination	gifta	aktiv
inblandade	framträdande	björk
fastigheter	dominerar	återkommer

javisst	behandlingen	förhållandena
behov	uppgörelse	ben
varv	fint	kamp
rummet	jobbar	exempelvis
upprepade	bekymmer	belopp
bortom	medlemskap	glömma
bekämpa	överens	farbror
åtminstone	vända	omvärlden
hemmaplan	universitet	bakåt
föreställningen	myndigheterna	arbetsmarknaden
manus	halvtimme	frivilligt
flest	omgivning	universum
huruvida	konservativa	komplicerade
programmet	kommersiella	motiv
förekommer	blå	ifrån
följa	perspektiv	valet
leva	blommor	medel
absolut	fara	emellertid
bedöma	rapport	beträffande
forna	berömda	förefaller
knapp	föräldrarna	öppnades
medverka	återvända	inträffade
arbetsgivarna	arbetstillfällen	kommissionen
egenskaper	informera	dramatiska
konsekvenserna	fånga	grupp

gammal	avtal	bestämma
grepp	framtiden	uppmärksamhet
mår	bilar	nummer
förbättra	stoppa	uppenbarligen
visas	möter	jämfört
magen	upplevelse	besvär
utbildning	minne	uppmanar
framhåller	effektivare	förändringar
byter	fåtal	form
framgångsrika	grov	hoppade
undervisning	experiment	läppar
nämna	effekterna	huvudstaden
film	brev	avgjorde

Appendix: B

Participant code: List order: Sound condition order:

Sex:

Age:

Below are some questions about how you experienced this part of the experiment. Please respond by ticking the scales.

1. How did you experience the sound condition? [*after SSN and 4TB only*]

|____|

Almost unbearable Not distracting at all

2. Why did you experience the sound condition this way? [*after SSN and 4TB only*]

3. How did the sound condition affect your performance, according to you? [*after SSN and 4TB only*]

|____|

Very negatively Very positively

4. Why did the sound condition affect your performance in this way? [*after SSN and 4TB only*]

5. How well do you consider that you performed?

|____|

Very poorly   Very well

6. How effortful was it for you to achieve your level of performance?

|____|

Very effortful   Not at all effortful

7. Did you use a strategy to perceive what was being said? If so, please describe it!

